# Reward processing in schizophrenia: a pilot fMRI study for examining delusions using the monetary incentive delay task

**DOI:** 10.1192/j.eurpsy.2025.2279

**Published:** 2025-08-26

**Authors:** A. Urkola, I. Gomez-Alonso, M. Á. García-León, A. Karuk, N. Ramiro, P. McKenna, P. Fuentes-Claramonte, E. Pomarol-Clotet

**Affiliations:** 1 FIDMAG Hermanas Hospitalarias Research Foundation; 2 Universitat de Barcelona, Barcelona; 3 Universidad de Sevilla, Sevilla; 4 Hospital de San Rafael; 5 CIBERSAM, Barcelona, Spain

## Abstract

**Introduction:**

Schizophrenia is a severe psychiatric disorder characterized by positive (delusions and hallucinations), negative and disorganization symptoms. According to the influential dopamine hypothesis, positive symptoms of schizophrenia are linked to increased dopamine transmission in subcortical regions, particularly the striatum. Kapur’s aberrant salience theory further suggests that hyperdopaminergia leads to increased and disorganized reward prediction error signalling, leading to the misattribution of significance to irrelevant stimuli, which contributes to the development of delusions. Negative symptoms have been argued to reflect reduced reward prediction error signalling.

**Objectives:**

To design an optimized monetary incentive delay (MID) task for use in fMRI studies of schizophrenia patients. For this, a pilot study was conducted in order to test the effects of monetary incentives on task performance in schizophrenia patients and healthy controls.

**Methods:**

Nine healthy controls and seven patients with DSM-5 schizophrenia completed the MID task, including a training and test phase. This task evaluates reward processing by presenting cues that predict either rewarding or non-rewarding outcomes before a target that requires a speeded response. We investigated the effect of these cues on task performance (accuracy and reaction times) through a repeated measures ANOVA that compared rewarding and non-rewarding cues with a between-subjects factor for diagnosis.

**Results:**

The patients with schizophrenia exhibited slower RT and lower accuracy compared to healthy controls (main effect of group, RT *p* = 0.008, accuracy *p* = 0.047). There was also a significant main effect of condition, with better accuracy and shorter RT in the rewarded condition in both the patients and controls (accuracy *p* = 0.01, RT *p* = 0.003). However, there was no significant group*condition interaction. Both the patients and the controls showed significant improvements in task performance when rewards were offered, compared to when no rewards were provided.

**Conclusions:**

Our MID task shows expected performance effects in patients with schizophrenia, producing effects comparable to those observed in healthy controls. This MID task design is therefore suitable for examining and understanding symptom profiles associated with reward processing, such as delusions and negative symptoms.

**Disclosure of Interest:**

None Declared

